# Characterization of Odontogenic Differentiation from Human Dental Pulp Stem Cells Using TMT-Based Proteomic Analysis

**DOI:** 10.1155/2020/3871496

**Published:** 2020-12-10

**Authors:** Xijuan Xiao, Caihong Xin, Yuqin Zhang, Jie Yan, Zhao Chen, Huiyong Xu, Min Liang, Buling Wu, Fuchun Fang, Wei Qiu

**Affiliations:** ^1^Yuncheng Stomatological Hospital, Yuncheng Stomatological Health School, South Section of Yuxi Road, Yuncheng 044000, China; ^2^Guangdong Provincial Key Laboratory of Oral Diseases, Guangzhou 510055, China; ^3^Department of Stomatology, Nanfang Hospital, Southern Medical University, 1838 Guangzhou Avenue North, Guangzhou 510515, China; ^4^Department of Periodontology, Guanghua School and Hospital of Stomatology and Guangdong Provincial Key Laboratory of Stomatology, Sun Yat-sen University, Guangzhou, Guangdong 510055, China

## Abstract

**Background:**

The repair of dental pulp injury relies on the odontogenic differentiation of dental pulp stem cells (DPSCs). To better understand the odontogenic differentiation of DPSCs and identify proteins involved in this process, tandem mass tags (TMTs) coupled with liquid chromatography-tandem mass spectrometry (LC-MS/MS) were applied to compare the proteomic profiles of induced and control DPSCs.

**Methods:**

The proteins expressed during osteogenic differentiation of human DPSCs were profiled using the TMT method combined with LC-MS/MS analysis. The identified proteins were subjected to Gene Ontology (GO) and Kyoto Encyclopedia of Genes and Genomes (KEGG) pathway analyses. Then, a protein-protein interaction (PPI) network was constructed. Two selected proteins were confirmed by western blotting (WB) analysis.

**Results:**

A total of 223 proteins that were differentially expressed were identified. Among them, 152 proteins were significantly upregulated and 71 were downregulated in the odontogenic differentiation group compared with the control group. On the basis of biological processes in GO, the identified proteins were mainly involved in cellular processes, metabolic processes, and biological regulation, which are connected with the signaling pathways highlighted by KEGG pathway analysis. PPI networks showed that most of the differentially expressed proteins were implicated in physical or functional interaction. The protein expression levels of FBN1 and TGF-*β*2 validated by WB were consistent with the proteomic analysis.

**Conclusions:**

This is the first proteomic analysis of human DPSC odontogenesis using a TMT method. We identified many new differentially expressed proteins that are potential targets for pulp-dentin complex regeneration and repair.

## 1. Introduction

The development of dental-derived mesenchymal stem cells is an intriguing milestone of regenerative medicine, in view of their capability of differentiating into osteogenic, adipogenic, and chondrogenic lineages, representing a promising source for the bone and dentin mineralization treatment strategies in the future [1]. Dental pulp stem cells (DPSCs), a group of dental-derived mesenchymal stem cells derived from the neural crest, are considered important seed cells in dental tissue engineering for pulp-dentin complex regeneration [2, 3]. When teeth are stimulated by dental caries, wear, or trauma, resident DPSCs migrate quickly to the injured site because of their suited location to secrete proregenerative cytokines to respond to the inflammatory microenvironment, then proliferate and differentiate into odontoblasts [4]. The formation of restorative dentin produced by odontoblasts could prevent disease progression to preserve dental pulp vitality [5, 6]. When new regenerated dentin tissue is well integrated into the previously damaged teeth, clinical healing occurs [7, 8]. This repair potential of dental pulp tissue provides a reliable biological basis for the study of pulp-dentin complex regeneration.

Proteomics can be used as an unbiased, global informatics tool to discover information about all protein expression levels and posttranslational modifications in cells or tissues [9]. The main quantitative techniques used in proteomics include gel-based proteomics (two-dimensional fluorescence difference gel electrophoresis (2D-DIGE), sodium dodecyl sulfate-polyacrylamide gel electrophoresis (SDS-PAGE)) and gel-free proteomics (mass spectrometry-based) [10–12]. Quantitative proteomics is crucial to understand the comprehensive protein expression profile underlying the molecular mechanisms of biological processes and disease states [13]. Most quantitative proteomic techniques involve the isotopic labeling of proteins or peptides in two or more experimental groups, which can then be differentiated by mass spectrometry. At present, the technologies of isobaric tags for relative and absolute quantitation (iTRAQ) and tandem mass tags (TMTs), chosen according to the sample number, are two widely used quantitative proteome labeling techniques [14, 15].

Wei et al. used 2D-DIGE and matrix-assisted laser desorption/ionization-time of flight mass spectrometry (MALDI-TOF-MS) technologies to explore the proteomic profile at the early stage (7 days) of odontogenic differentiation in dental pulp cells (DPCs). Twenty-three proteins were screened out in their study. The expression of heterogeneous nuclear ribonucleoprotein C, annexin VI, collagen type VI, and matrilin-2 was validated by quantitative real-time polymerase chain reaction (qRT-PCR) and western blotting (WB) [16]. In 2013, Kim et al. analyzed the secretome of human DPSCs after 3 days of odontogenic differentiation using SDS-PAGE/LC-MS/MS. The protein lysyl oxidase-like 2 (LOXL2) inhibited the odontogenic differentiation of DPSCs [17]. Gel-based techniques were applied in the above two studies. However, the gel-based techniques' low sensitivity, poor separation, and poor resolution for particular types of proteins and their lack of accuracy for an individual protein within a mixed spot undermine their prospects for profound and accurate proteomic research [18]. As an alternative, gel-free quantitative proteomics with greater accuracy and sensitivity is needed for studies about the protein profile of DPSCs during odontogenic differentiation.

Our study is the first investigation of proteomic profiles in the process of odontogenic differentiation of human DPSCs using TMT combined with LC-MS/MS and provides further insight into the molecular mechanisms in reparative dentinogenesis.

## 2. Materials and Methods

### 2.1. DPSC Isolation, Culture, and Identification

Healthy and intact premolars were extracted from 23 healthy individuals (13 females and 10 males in the 15-25 age range, mean age of 19.7) who were receiving orthodontic treatment at the Department of Stomatology, Nanfang Hospital, Southern Medical University. Teeth had been collected from April to December 2019. This project was approved by the Ethics Committee of Nanfang Hospital, Southern Medical University. DPSCs isolated from the pulp tissue of these premolars were cultured in routine media as we described previously [19].

DPSCs were identified by flow cytometry (Becton Dickinson, Tokyo, Japan). hDPSCs were stained with anti-phycoerythrin (PE), anti-fluorescein isothiocyanate (FITC), anti-CD44-FITC, anti-CD29-PE, anti-CD45-PE, and anti-CD34-PE (BD Pharmingen, Franklin Lakes, NJ) antibodies. Isotype-identical antibodies served as controls. All procedures were carried out according to the manufacturer's instructions [20].

### 2.2. Odontogenic Induction

DPSCs were induced with an odontogenic differentiation medium which contains 100 nmol/L dexamethasone, 50 mg/mL ascorbic acid, and 10 mmol/L *β*-glycerophosphate (Sigma-Aldrich, St. Louis, MO, USA) in 6-well plates. DPSCs in the noninduced group were cultured in the DMEM+10% FBS. After 14-day culture, the cells were stained with Alizarin Red S (ARS A5533, Sigma-Aldrich). We observed and photographed the calcium nodules with a microscope (Crystal Violet, Amresco, Solon, OH). ALP staining was performed after 7 days of culture in the odontogenic differentiation medium following the protocol of the NBT/BCIP Staining Kit (Beyotime Biotechnology, Shanghai, China).

### 2.3. Preparation of Protein Samples

Induced DPSCs were cultured for 14 days, and SDT lysate (4% SDS, 100 mM Tris-HCl, 1 mM DTT, and pH 7.6) was added. After ultrasound (80 W, 10 s per operation, 15 s intermittency, and 10 cycles), the cell lysates were bathed at 100°C for 15 min and then centrifuged at 14,000g for 40 min. The supernatant was kept, and a BCA kit was used for protein quantification.

### 2.4. SDS-PAGE Separation

Twenty micrograms of protein was taken from each sample, and 5x loading buffer (10% SDS, 0.5% bromophenol blue, 50% glycerol, 500 mM DTT, 250 mM Tris-HCl, and pH 6.8) was added. 12.5% SDS-PAGE electrophoresis (constant current 14 mA, 90 min) was performed after 5 min of boiling in a water bath, and the gel was then stained with Coomassie blue.

### 2.5. Filter-Aided Sample Preparation (FASP Digestion)

Thirty microliters of protein solution was taken from each sample. DTT (100 mM) was added separately, and the solution was cooled to room temperature after 5 min in a boiling water bath. We added 200 *μ*L UA buffer (8 M urea, 150 mM Tris-HCl, and pH 8.0) and mixed it well, then transferred it to a 10 kD ultrafiltration centrifuge tube, centrifuged the tube at 14,000g for 15 min, discarded the filtrate, and repeated this centrifugation once. We added 100 *μ*L IAA buffer (100 mM IAA in UA), oscillated the sample at 600 rpm for 1 min, let it react at room temperature in the dark for 30 min, and centrifuged it at 14,000g for 15 min. We added 10 *μ*L UA buffer and centrifuged the sample at 14,000g for 15 min. This step was repeated twice. We next added 100 *μ*L of 100 mM TEAB buffer and centrifuged the sample at 14,000g for 15 min. This step was also repeated twice. After 40 *μ*L trypsin buffer (4 *μ*g trypsin in 40 *μ*L 100 mM TEAB buffer) was added, the sample was oscillated at 600 rpm for 1 min and placed at 37°C for 16-18 h. The collection tube was replaced, and the tube was centrifuged at 14,000g for 15 min. Then, 40 *μ*L of 10-fold diluted 100 mM TEAB buffer was added, and the sample was centrifuged at 14,000g for 15 min. The filtrate was collected, and the peptide was quantified for its OD280.

### 2.6. TMT Labeling

Each sample was labeled with 100 *μ*g of peptide fragments according to the manufacturer's instructions for the TMT labeling kit (Thermo Fisher Scientific, Waltham, MA, USA). Peptides of the two groups were labeled with different TMTs: three biological repeats of the control group were labeled with TMT-126, TMT-127, and TMT-128, respectively, and three biological repeats of the exercise group were labeled with TMT-129, TMT-130, and TMT-131, respectively.

### 2.7. Peptide Fractionation

After mixing the labeled peptide segments of each group in equal amounts, classification was performed using a high-pH RP spin column. After peptide labels were mixed and lyophilized, 100 *μ*g was diluted with 300 *μ*L of 0.1% trifluoroacetic acid and transferred to a high-pH RP spin column. The FT component was collected centrifugally, 300 *μ*L of pure water was added, the wash component was collected centrifugally, and step gradient elution was started. After freeze-drying, the sample was redissolved with 12 *μ*L of 0.1% formic acid, and the peptide concentration was calculated by determining the OD280.

### 2.8. High-Performance Liquid Chromatography (HPLC) and LC-MS/MS Analysis

Each fraction was injected for nano-LC-MS/MS analysis. Each sample was separated by a high-performance liquid-phase system, EASY-nLC with a nanoliter flow rate. The chromatographic column was balanced with 95% buffer A (0.1% formic acid aqueous solution). The sample was loaded onto the loading column (Thermo Scientific Acclaim PepMap 100, 100 *μ*m × 2 cm, Nanoviper C18) by an automatic sampler and then separated by an analysis column (Thermo Scientific EASY-Column, 10 cm, ID75 *μ*m, 3 *μ*m, C18-A2) at a flow rate of 300 nL/min by IntelliFlow technology.

Samples were separated by liquid chromatography and analyzed by a Q Exactive mass spectrometer. The analysis duration was 60/90 min, the positive ion mode was used for detection, the scanning range of the parent ions was 300-1800 *m*/*z*, the primary mass spectrum resolution was 70,000 at 200 *m*/*z*, the AGC target was 3e6, the primary maximum IT was 10 ms, the number of scan ranges was 1, and the dynamic exclusion was 40 s. The mass-to-charge ratio of polypeptides and polypeptide fragments were determined according to the following methods: 10 fragment patterns (MS2 scan) were collected after each full scan, the MS2 activation type was HCD, the isolation window was 2 *m*/*z*, the secondary mass spectrum resolution was 17,500 at 200 *m*/*z* (TMT6plex) or 35,000 at 200 *m*/*z* (TMT10plex), there was 1 microscan, the secondary maximum was 60 ms, the normalized collision energy was 30 eV, and the underfill was 0.1%.

### 2.9. Protein Identification and Quantitative Analysis

MS/MS spectra were searched using the MASCOT engine (Matrix Science, London, UK; version 2.2) embedded into Proteome Discoverer 1.4. The search criteria were set as follows: all tryptic specificity was required; 2 missed cleavages were allowed; carbamidomethylation (C), TMT6plex (N-terminal), and TMT6plex (lysine, K) were set as the fixed modifications; oxidation (methionine, M) and TMT6plex (tyrosine, Y) were set as the variable modifications; peptide mass tolerances were set at 20 ppm for all MS1 spectra acquired; and fragment mass tolerances were set at 0.1 Da for all MS2 spectra acquired. The peptide false discovery rate (FDR) was set as ≤0.01. All peptide ratios were normalized by the median protein ratio. The thresholds were set at the ratio of exercise/control ≥ 1.2 and *p* value ≤ 0.05 for upregulation. Similarly, the thresholds were set at the ratio of exercise/control ≤ 0.83 and *p* value ≤ 0.05 for downregulation (refer to previous studies [21, 22]).

### 2.10. Gene Ontology (GO) Function Notes

The process of GO annotation of the target proteins set by Blast2GO can be roughly summarized into four steps: sequence alignment, GO item extraction, GO annotation, and supplementary annotation. First, the protein sequences of differentially expressed proteins (FASTA format) were retrieved in batches from the UniProtKB database (version 2016_10). NCBI BLAST client software (ncbi-blast-2.2.28-win32.exe) was used to carry out a local search on the retrieved sequences to find the homologous sequence neural network annotations. In this work, the first 10 BLAST values of each query sequence were retrieved if they were less than 1*e* − 3, and they were loaded into Blast2GO10 (version 3.3.5) for GO mapping and annotation. In the annotation process, the Blast2GO Command Line annotates the GO entries extracted in the entry extraction process to the target protein sequence by comprehensively considering the similarity between the target protein sequence and the alignment sequence, the reliability of the source of the GO entries, and the structure of the GO directed acyclic graph. After the annotation was completed, in order to further improve the annotation efficiency, conserved motifs found in the target protein sequence in the EBI database were searched through InterProScan, and the functional information related to the motifs was annotated to the target protein sequence. ANNEX was run to further supplement the annotation information, and links were established between different GO categories to improve the annotation accuracy. For each category, a two-tailed Fisher exact test was employed to test the enrichment of the differentially expressed protein against all identified proteins. The GO with a corrected *p* value < 0.05 is considered significant.

### 2.11. KEGG (Kyoto Encyclopedia of Genes and Genomes) Pathway Notes

In the KEGG database, KO is the classification system of genes and their products. Orthologous genes with similar functions in the same pathway and their products are divided into a group, and the same KO (or K) marker is applied to them. When carrying out the KEGG pathway annotation on the target proteome, KASS (KEGG Automatic Annotation Server) software was first used to compare the target proteome with the KEGG GENES database. The target proteome sequence was KO-classified, and the path information related to the target proteome sequence was automatically obtained based on the KO classification. The results were filtered by the following criteria: a corrected *p* value < 0.05 and protein counts > 5.

### 2.12. Protein Interaction Network Analysis

First, the gene symbol of each target protein was obtained from the source database of the target protein sequence, and then the gene symbol was put into the IntAct (http://www.ebi.ac.uk/intact/main.xhtml) or STRING (http://string-db.org/) database. The protein-protein interaction (PPI) networks of the differentially expressed proteins were established based on STRING (Search Tool for the Retrieval of Interacting Genes). We set a confidence score ≥ 0.4 and the maximum number of interactors = 0 as the cutoff criterion. Then, the interaction network of the differentially expressed proteins was screened by the cytoHubba from the Cytoscape software 3.2.1 (http://www.cytoscape.org/) platform according to the high degree of connectivity [23].

### 2.13. Western Blotting

Cells were lysed in RIPA buffer (Beyotime, Nanjing, China) supplemented with protease inhibitors. Protein samples were separated by SDS-PAGE in a 15% gel and transferred to polyvinylidene difluoride membranes (Amersham, Little Chalfont, UK) at 200 mA for 2-3 hours. The membranes were blocked with 5% skim milk for 1 hour and incubated with primary antibody overnight at 4°C. Antibodies against Fibrillin-1 (FBN1), transforming growth factor-*β*2 (TGF-*β*2), and *β*-actin were purchased from Shanghai Applied Protein Technology. *β*-Actin was the internal loading control. After washing with Tris-buffered saline containing 0.05% Tween 20 (TBS-T) three times, samples were incubated with the secondary horseradish peroxidase-conjugated antibody (Proteintech, China). Immunoreactive proteins were visualized by using an ECL Kit (Beyotime Biotechnology, Shanghai, China).

### 2.14. Statistical Analysis

Band intensity in WB images was quantified with ImageJ software. Each data point is expressed as the mean ± standard deviation (SD), and the assay was repeated at least three times. Statistical analysis was performed by the *t*-test and one-way ANOVA using SPSS 17.0 for Windows (SPSS, Chicago, IL, USA). Statistical significance was defined as *p* < 0.05.

## 3. Results

### 3.1. Characteristics of Human DPSCs

**Figure 1:**
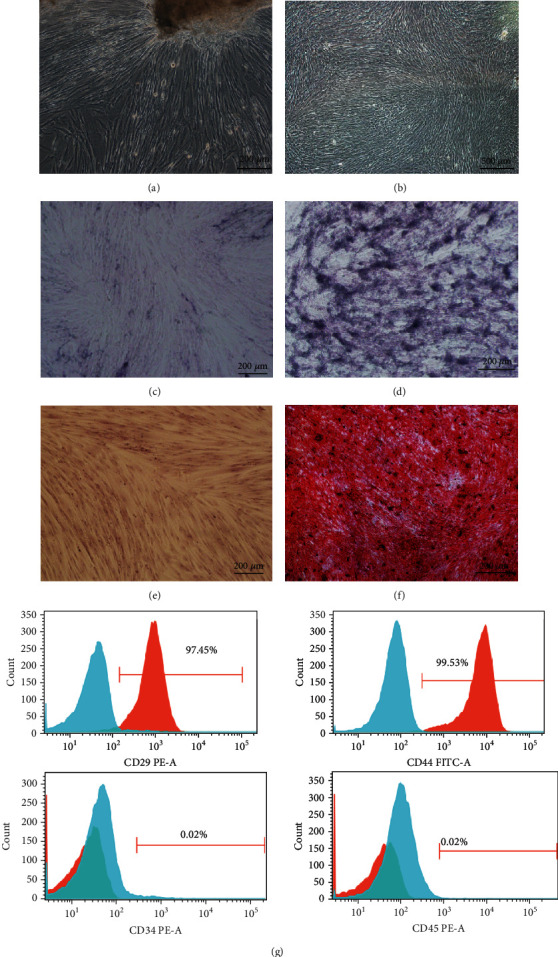
Culture, isolation and identification of human DPSCs. (a, b) Primary cultured DPSCs. (c–f) Odontogenic differentiation of DPSCs was assessed by ALP and Alizarin Red S staining. (g) Flow cytometry was used to detect the surface markers of DPSCs. Cells were incubated with fluorescence-conjugated antibodies against CD29, CD34, CD44, and CD45. Isotype-identical antibodies served as controls. Analysis of surface antigens in DPSCs by flow cytometry indicated that the cells were positive for CD29 and CD44, while CD34 and CD45 were negative (red line).

Cells emerged from the tissue bulk adhering to the dish and preformed obvious fibroblast-like morphology (Figure 1(a)) after 14 days of culture. Using a limited dilution technique, we obtained the DPSCs (Figure 1(b)). The protein level of ALP increased with a rapid increase after 7 days of odontogenic induction (Figure 1(d)) compared with the control group (Figure 1(c)). After 14 days of induction, mineralized nodules were seen in the induced group by ARS staining (Figure 1(f)), but not in the control group (Figure 1(e)). Flow cytometry was used to determine mesenchymal stem cell surface markers (CD29 and CD44) and hematopoietic cell markers (CD34 and CD45). The cells were identified to be positive for CD29 and CD44 and negative for CD34 and CD45 (Figure 1(g)), indicating the mesenchymal lineage of hDPSCs.

### 3.2. Differentially Expressed Protein Profile

**Figure 2:**
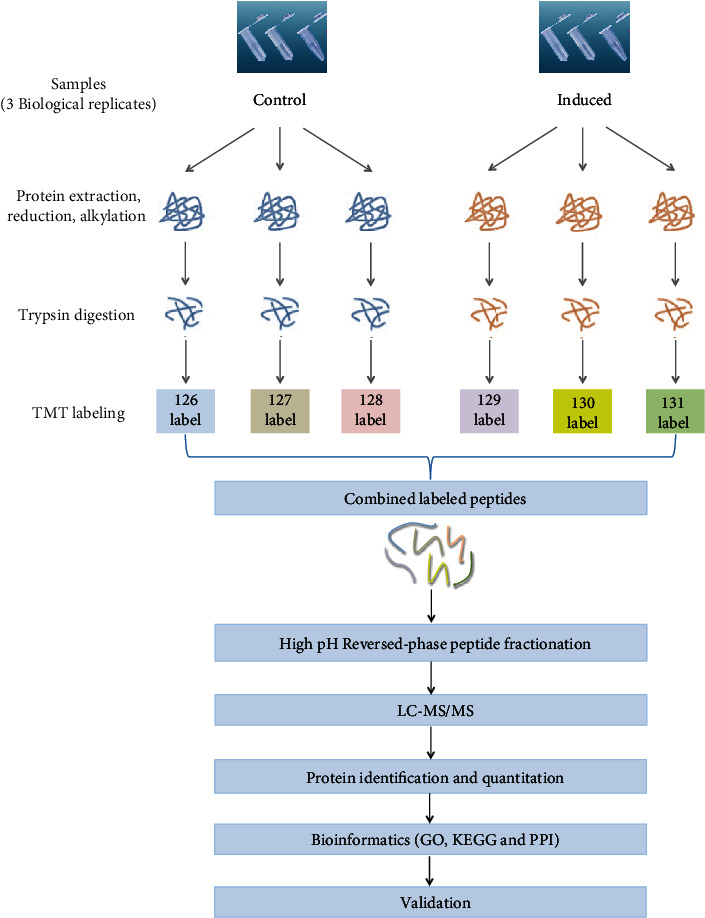
Flow diagram of the TMT-based quantitative proteomic platform applied to identify proteomic profiles. DPSCs were induced for 14 days or not, and whole cellular proteins were extracted from the two groups and quantified. Following trypsin digestion of equal amounts of protein, the resolved peptides were labeled with TMT6plex reagents, fractionated by HPLC, and analyzed by LC-MS/MS.

**Figure 3:**
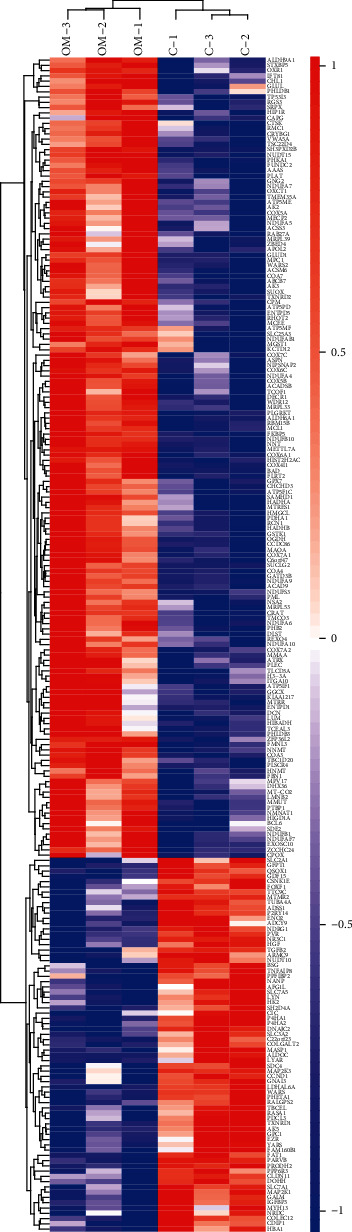
DPSC odontogenesis proteome. Differentially expressed proteins were analyzed by hierarchical clustering. Heat map indicates that expression patterns varied among different groups. Red indicates a high relative expression level, whereas blue indicates a low relative expression level.

To get an overview of the data, the expression of endogenous proteins in three induced groups and three control groups was analyzed using a TMT-based quantitative proteomic approach. A flow diagram of the TMT-based quantitative proteomic platform applied to identify proteomic profiles is shown in Figure 2. A total of 223 proteins that were differentially expressed between the induced and control DPSC groups were identified using TMT analysis and are shown in Tables S1 and S2. Hierarchical clustering showed that the expression levels of proteins in the differentiated group differed significantly from those in the undifferentiated group according to the fold change (greater than 1.2 or less than 0.83) and *p* value thresholds (less than 0.05). Among these, 152 proteins were upregulated and 71 were downregulated (Figure 3). Tables 1 and 2 list the top 20 upregulated and downregulated proteins.

### 3.3. Functional Classification of the Differentially Expressed Proteins

**Figure 4:**
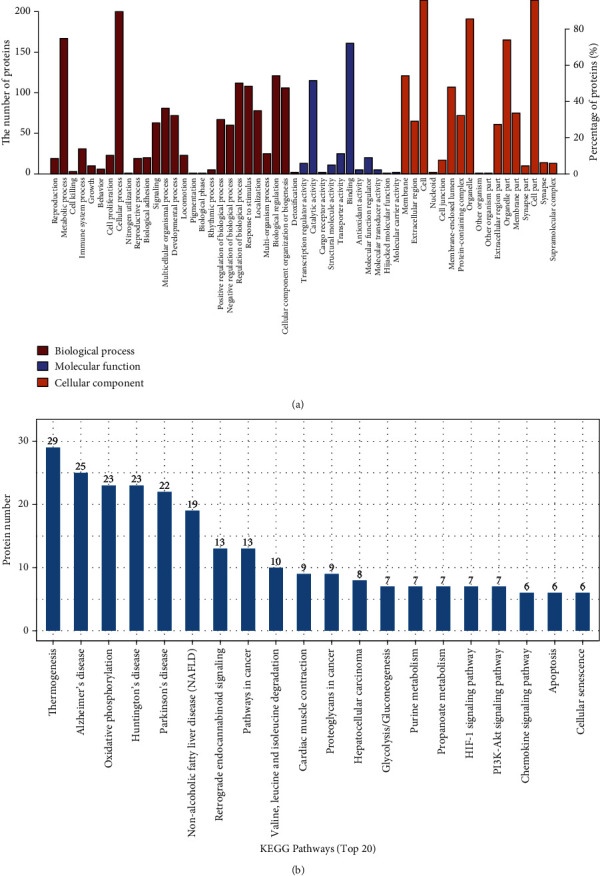
GO and KEGG pathway analyses. (a) GO classification of differentially upregulated and downregulated proteins in DPSCs during odontogenic differentiation. (b) KEGG pathway analysis of differentially expressed proteins in DPSCs during odontogenic differentiation.

GO analysis with the assistance of DAVID Bioinformatics Resources was conducted to identify the functions of proteins identified using the TMT technique. The detailed functional classifications of the differentially expressed proteins are shown in Figure 4(a). Briefly, the classification by biological processes showed that the proteins were mainly involved in cellular processes, metabolic processes, biological regulation, regulation of biological processes, responses to stimuli, cellular component organization, and biogenesis (>40% for each class). On the basis of molecular function, the proteins in our study were implicated in binding, catalytic activity, transporter activity, molecular function regulator, transcription regulator activity, etc. In the cellular component ontology, we found that the majority of enriched categories were associated with the cell, cell part, organelle, organelle part, membrane, etc.

We then performed KEGG analysis to investigate the enriched pathways that the differentially expressed proteins participated in during odontogenic differentiation. We found that a total of 223 altered proteins could be mapped to 238 signaling pathways (*p* < 0.05) (Table S3). The top enriched pathways of the altered proteins were thermogenesis, Alzheimer's disease, oxidative phosphorylation, etc.

### 3.4. Protein Interaction Network Analysis

**Figure 5:**
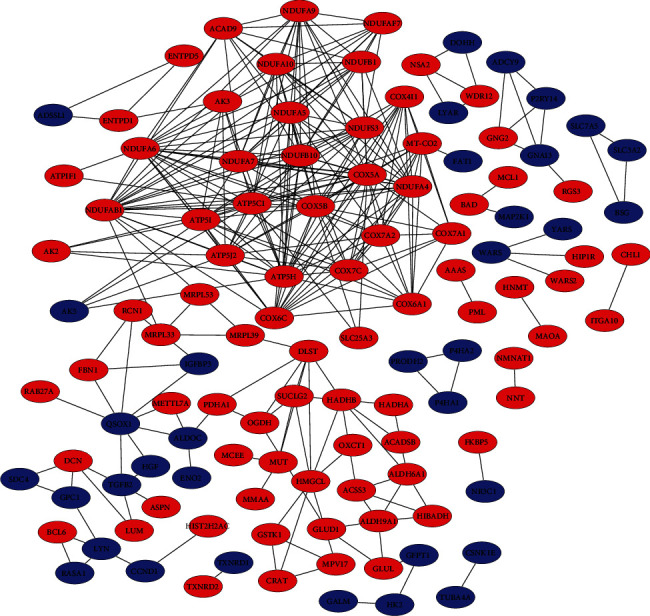
The protein-protein interaction network of differentially expressed proteins. A node represents a protein, and a line represents an interaction between two proteins. The red and blue nodes in the network are significantly upregulated and downregulated proteins, respectively.

STRING database analysis was used to build a protein-protein interaction (PPI) network concerning the process of odontogenic differentiation in DPSCs. Most of the differentially expressed proteins were implicated in physical or functional interaction. In this PPI network, we found that 223 proteins were mapped to 14 known protein-protein interaction networks, and 22 proteins had an interaction score of more than ten (Table 3). Among these proteins, cytochrome c oxidase subunit 5A (COX5A) was the most vital hub, interacting with 23 proteins. FBN1 and TGF-*β*2, which were reported to be involved in odontogenesis, were present in the most complex networks (Figure 5).

### 3.5. Western Blotting Validation

**Figure 6:**
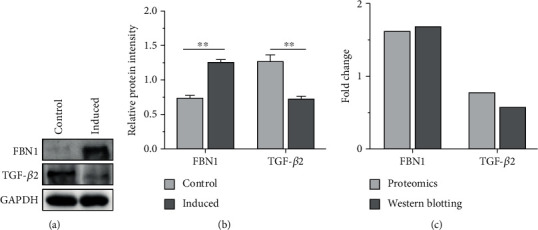
Validation results of western blotting. (a, b) Western blotting analysis confirmed the upregulation of FBN1 and the downregulation of TGF-*β*2 14 days after odontogenic induction of DPSCs. (c) Heights of the columns in the chart represent mean fold changes in the expression of FBN1 and TGF-*β*2. The validation results were consistent with the proteome data.

Two differentially expressed proteins, FBN1 and TGF-*β*2, involved in odontogenesis were selected and validated using western blotting. We found that the levels of FBN1 and TGF-*β*2 in induced cells were increased approximately 1.69-fold and decreased approximately 0.58-fold, respectively (Figures 6(a) and 6(b)). These validation results were consistent with the protein analysis data (Figure 6(c)).

## 4. Discussion

To date, there are only two reports concerning the proteomic profile involving odontogenesis, and they have taken traditional gel-based proteomic approaches [16, 17]. Wei et al. identified 23 differentially expressed proteins related to the early odontogenic differentiation of DPSCs using 2-DE coupled with MS [16]. Kim et al. found that LOXL2 protein was downregulated and had a negative effect in the hDPSCs that differentiate into odontoblast-like cells using gel trypsin digestion coupled with LC-MS/MS proteomic approaches [17]. Although 2-DE proteomic strategies provided the first insight into the proteomic landscape of DPCs during odontogenic differentiation at an early stage, they also have a number of serious limitations, such as the inability to isolate acidic, basic, and hydrophobic (membrane) proteins and a limited number of obtained proteins [24]. To overcome these limitations, we applied, for the first time, advanced gel-free nano-LC-MS/MS technology to characterize the full proteome of DPSCs during odontogenic differentiation. TMT is an in vitro polypeptide labeling technique developed by Thermo Fisher Scientific. By using multiple isotope tags and covalent binding reactions with amino groups of peptides, this technique can achieve the qualitative and quantitative analyses of proteins in 2, 6, or 10 different samples at the same time. It has the advantages of accurate quantification, good repeatability, and high sensitivity. Therefore, it is widely used in the analysis of differentially expressed proteins [13, 25]. In this study, we tried to explore the molecular basis of dentin differentiation through proteomic methods based on TMT technology. A total of 223 proteins were differentially expressed during odontogenic differentiation of DPSCs, which far exceeded the number of differentially expressed proteins identified in the above two studies.

Pleckstrin homology-like domain family B member 3 (PHLDB3) was the most upregulated protein (fold change: 8.93) among the differentially expressed proteins. PHLDB3 was once thought to be a tumor suppressor. Recent research found that PHLDB3 could increase tumor growth by inactivating p53 via a negative feedback loop in pancreatic, prostate, colon, breast, lung, and other common cancers [26]. There are few reports on PHLDB3 in cellular differentiation, and the potential role of PHLDB3 in odontogenic differentiation needs more research. The lowest-expressed protein was the cell growth-regulating nucleolar protein Ly1 antibody reactive (LYAR). LYAR is a zinc finger nucleolar protein that has been implicated in cell growth, self-renewal of ESCs, and medulloblastoma [27, 28]. Li et al. reported that it is highly expressed in undifferentiated ESCs and plays a critical role in maintaining ESC identity. The reduced expression of LYAR in ESCs impairs their differentiation capacity [29]. The regulatory role of LYAR in ESC differentiation indicates that it might function in the odontogenic differentiation of DPSCs.

It was noted that there were significant differences in the expression of some proteins that are involved in the process of odontogenic differentiation, including FBN1 (upregulated fold change: 1.62) and TGF-*β*2 (downregulated fold change: 0.77). FBN1 was proven to be a key molecule forming the backbone of microfibrils [30]. More evidence has revealed that FBN1 plays an important role in the extracellular regulation of TGF-*β* as well as bone morphogenetic protein (BMP) activation and signaling, which are essential for odontogenic differentiation and reparative dentinogenesis [31]. Yoshiba et al. found that FBN1 upregulation was accompanied by wound healing in dental pulp tissue [32]. Our previous study found that the mRNA and protein expression of FBN1 was increased during the odontogenic differentiation of DPSCs, and the lncRNA-G043225/miR-588/FBN1 axis was involved in the odontogenic differentiation of DPSCs [19]. TGF-*β*2 was also identified to be an important regulator of DPSC differentiation [33]. Yu et al. induced the odontogenic differentiation of stem cells from dental apical papilla (SCAPs) and bone marrow (BMSCs) and tracked the expression of secretory proteins during early odontogenic differentiation using TMT combined with HPLC-MS/MS analysis [34]. The results revealed that TGF-*β*2 was significantly upregulated during the odontogenic differentiation of SCAPs and was significantly downregulated during the odontogenic differentiation of BMSCs. Tai et al. found that TGF-*β*2 possibly regulates the differentiation of pulp cells via an autocrine fashion by activation of the ALK/Smad2/3 signal transduction pathways at specific stages synergistically with other factors [33]. In our study, TGF-*β*2 was significantly downregulated during the odontogenic differentiation of DPSCs. Thus, we can conclude that TGF-*β*2 is a potentially important molecule with a distinct function in the regulation of odontogenesis. The exact regulatory mechanism of TGF-*β*2 in the odontogenic differentiation of DPSCs needs further in-depth research.

According to our GO analysis, the functions of proteins identified using the TMT technique included cellular processes, metabolic processes, binding, and catalytic activity, which were directly or indirectly related to cell differentiation. Functional annotation clustering and pathway analysis showed that oxidative phosphorylation, hypoxia-inducible factor-1 (HIF-1) signaling, and PI3K-Akt signaling were in the top 20 pathways. These three signaling pathways have been identified to regulate osteogenic/odontogenic differentiation through different underlying mechanisms [35–38].

Studying the interaction between proteins and the network formed by their interaction is of great significance to reveal the functions of proteins [39, 40]. In PPI networks, proteins that interact directly with many other proteins are called hubs. A greater number of hubs indicate more importance to the whole system. Proteins with more interaction partners may play a key role in maintaining the balance and stability of the system, and they may be candidates for follow-up research [41]. In the PPI network constructed here, COX5A was the most vital hub. COX5A is a nuclear-encoded subunit of the terminal oxidase involved in mitochondrial electron transport [42]. Previous research indicated that the dysregulation of COX5A significantly affects COX function, thereby causing mitochondrial dysfunction in skeletal muscle, pulmonary arterial hypertension, lactic academia, and central nervous system diseases [43, 44]. COX5A, as an enzyme involved in oxidative phosphorylation, may play an important role in the odontogenic differentiation of DPSCs. However, little information has been reported about the role of COX5A in odontogenesis. The function and regulatory mechanism of COX5A in the odontogenic differentiation of DPSCs require further exploration.

The potential of odontogenic differentiation of DPSCs plays a crucial role in pulp-dentin complex regeneration in future clinical applications [2, 3]. In our study, DPSCs were cultured with the odontogenic medium supplementing 10% FBS. However, the clinical application of DPSCs in regenerative medicine demands an in vitro expansion and in vivo delivery, which must deal with the biological safety issues about animal serum in the unique cell model. Marrazzo et al. reported a highly efficient in vitro reparative behavior of DPSCs cultured with platelet lysate. This novel model could apply platelet lysate as a valid candidate for FBS to culture and osteogenic-differentiate DPSCs [45].Therefore, we would like to refer to Marrazzo's protocol and establish an in vitro differentiation cell model for furtherly exploring the clinical application of DPSCs in regenerative medicine.

## 5. Conclusions

Our study is the first to identify differentially expressed proteins related to the odontogenic differentiation of DPSCs using the TMT-based quantitative proteomic technique. Bioinformatics analyses suggest that a total of 223 proteins were differentially expressed during odontogenic differentiation of DPSCs and were mainly involved in cellular processes, metabolic processes, and biological regulation-related signaling pathways. Furthermore, FBN1 and TGF-*β*2, associating the odontogenic differentiation of MSCs, were confirmed to be differentially expressed, representing the potential regulation in the odontogenesis of hDPSCs. Our findings will facilitate a better understanding of the mechanisms of odontogenesis and provide a new perspective for research on pulp-dentin complex regeneration and repair.

## Figures and Tables

**Table 1:** Top 20 upregulated proteins in DPSCs during odontogenic differentiation.

Protein IDs^#^	Gene name	Description	FC	*p* value
Q6NSJ2	PHLDB3	Pleckstrin homology-like domain family B member 3	8.929617	0.042143712
Q9H8H3	METTL7A	Methyltransferase-like protein 7A	2.378968	0.000017
Q13451	FKBP5	Peptidyl-prolyl *cis*-*trans* isomerase FKBP5	2.091789	0.000634914
Q9H6F5	CCDC86	Coiled-coil domain-containing protein 86	2.058944	0.007063245
Q9NRG9	AAAS	Aladin	2.000935	0.006565251
Q9Y5U8	MPC1	Mitochondrial pyruvate carrier 1	1.953321	0.009136568
P49796	RGS3	Regulator of G-protein signaling 3	1.863234	0.018650261
P24310	COX7A1	Cytochrome c oxidase subunit 7A1, mitochondrial	1.834287	0.013307141
O43155	FLRT2	Leucine-rich repeat transmembrane protein FLRT2	1.825651	0.00393819
Q9Y3Z3	SAMHD1	Deoxynucleoside triphosphate triphosphohydrolase SAMHD1	1.694973	0.007160665
Q16777	HIST2H2AC	Histone H2A type 2-C	1.651296	0.004166831
P84243	H3-3A	Histone H3.3	1.63997	0.021062283
P35555	FBN1	Fibrillin-1	1.621603	0.040616831
P51687	SUOX	Sulfite oxidase, mitochondrial	1.601012	0.033377572
P12074	COX6A1	Cytochrome c oxidase subunit 6A1, mitochondrial	1.578785	0.000227675
P49961	ENTPD1	Ectonucleoside triphosphate diphosphohydrolase 1	1.565717	0.040866822
P59768	GNG2	Guanine nucleotide-binding protein G(I)/G(S)/G(O) subunit gamma-2	1.558081	0.015719252
Q9HAN9	NMNAT1	Nicotinamide/nicotinic acid mononucleotide adenylyltransferase 1	1.553307	0.011652959
Q6P461	ACSM6	Acyl-coenzyme A synthetase ACSM6, mitochondrial	1.550014	0.004336177
Q969E4	TCEAL3	Transcription elongation factor A protein-like 3	1.542699	0.027672968

^#^Protein codes from the UniProt database (http://www.uniprot.org). FC = fold change.

**Table 2:** Top 20 downregulated proteins in DPSCs during odontogenic differentiation.

Protein IDs^#^	Gene name	Description	FC	*p* value
Q9NX58	LYAR	Cell growth-regulating nucleolar protein	0.376297	0.043799582
Q8WV93	AFG1L	AFG1-like ATPase	0.540563	0.036347064
Q13614	MTMR2	Myotubularin-related protein 2	0.546523	0.015608944
P14210	HGF	Hepatocyte growth factor	0.548717	0.01763124
O15460	P4HA2	Prolyl 4-hydroxylase subunit alpha-2	0.554447	0.005722537
Q5W0V3	FAM160B1	Protein FAM160B1	0.565485	0.036798646
Q15391	P2RY14	P2Y purinoceptor 14	0.572628	0.008883045
O75508	CLDN11	Claudin-11	0.58844	0.020497794
Q9UF12	PRODH2	Hydroxyproline dehydrogenase	0.605641	0.000370822
Q01650	SLC7A5	Large neutral amino acids transporter small subunit 1	0.605646	0.039630129
Q9UKX3	MYH13	Myosin-13	0.63026	0.038419456
Q9BZE7	C22orf23	UPF0193 protein EVG1	0.631004	0.046583774
P15151	PVR	Poliovirus receptor	0.64692	0.006144973
P09104	ENO2	Gamma-enolase	0.649144	0.012865081
P69905	HBA1	Hemoglobin subunit alpha	0.668426	0.016508348
Q14517	FAT1	Protocadherin Fat 1	0.674727	0.004574163
P11166	SLC2A1	Solute carrier family 2, facilitated glucose transporter member 1	0.679885	0.034184575
Q96RK0	CIC	Protein capicua homolog	0.684228	0.046815763
Q9H305	CDIP1	Cell death-inducing p53-target protein 1	0.685535	0.041525227
O95379	TNFAIP8	Tumor necrosis factor alpha-induced protein 8	0.689731	0.00960706

^#^Protein codes from the UniProt database (http://www.uniprot.org). FC = fold change.

**Table 3:** The proteins with interaction degrees greater than ten in the PPI network.

Protein ID	Gene name	Degree
P20674	COX5A	23
O75947	ATP5PD	22
P56556	NDUFA6	21
P10606	COX5B	21
O14561	NDUFAB1	20
O96000	NDUFB10	19
O00483	NDUFA4	19
Q16718	NDUFA5	18
P36542	ATP5F1C	18
O75489	NDUFS3	18
P09669	COX6C	16
P56385	ATP5ME	16
P56134	ATP5MF	16
O95182	NDUFA7	15
Q16795	NDUFA9	15
P15954	COX7C	14
O95299	NDUFA10	14
P14406	COX7A2	13
P13073	COX4I1	12
O75438	NDUFB1	12
P00403	MT-CO2	11
P12074	COX6A1	11

## Data Availability

The mass spectrometry proteomic raw data have been deposited to the ProteomeXchange Consortium via the PRIDE [46] partner repository with the dataset identifier PXD021279. The data supporting the research results can be obtained from the corresponding authors according to reasonable requirements.

## Supplementary Materials

Supplementary 1 Table 1: details of all differentially upregulated proteins.

Supplementary 2 Table 2: details of all differentially downregulated proteins.

Supplementary 3 Table 3: all the mapped signaling pathways of the differentially expressed proteins.
