# Correction to “Characterization of Odontogenic Differentiation from Human Dental Pulp Stem Cells Using TMT‐Based Proteomic Analysis”

**DOI:** 10.1155/bmri/9797869

**Published:** 2026-05-22

X. Xiao, C. Xin, Y. Zhang, et al., “Characterization of Odontogenic Differentiation from Human Dental Pulp Stem Cells Using TMT‐Based Proteomic Analysis,” *BioMed Research International*, 2020, 3871496, 10.1155/2020/3871496

In the article, the authors have identified errors in panels A and E presented in Figure 1. Specifically, panel E of Figure 1 appears to be identical to Figure 9d in the author’s previous work [1]. As the current panel A did not arise from the same experiments as the correct panel E, panel A has been replaced for consistency. The correct Figure 1 is shown below:

**Figure 1:**
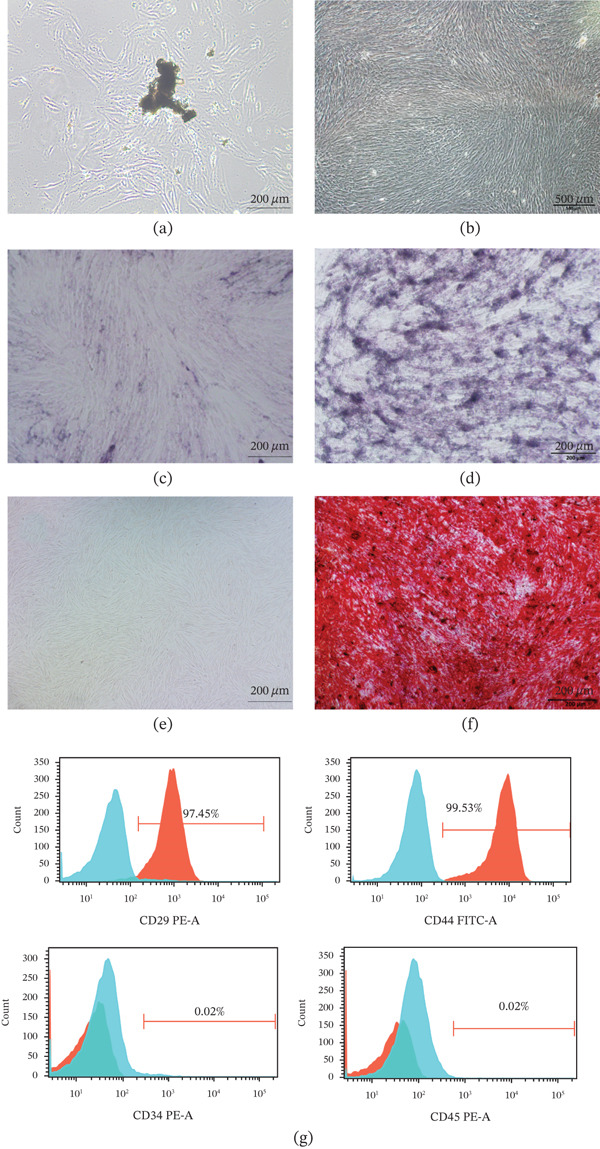
Culture, isolation, and identification of human DPSCs. (a, b) Primary cultured DPSCs. (c–f) Odontogenic differentiation of DPSCs was assessed by ALP and Alizarin Red S staining. (g) Flow cytometry was used to detect the surface markers of DPSCs. Cells were incubated with fluorescence‐conjugated antibodies against CD29, CD34, CD44, and CD45. Isotype‐identical antibodies served as controls. Analysis of surface antigens in DPSCs by flow cytometry indicated that the cells were positive for CD29 and CD44, whereas CD34 and CD45 were negative (red line).

We apologize for these errors.
